# Relationship between *Helicobacter pylori* Infections in Diabetic Patients and Inflammations, Metabolic Syndrome, and Complications

**DOI:** 10.1155/2015/290128

**Published:** 2015-01-22

**Authors:** Yusuf Kayar, Özgül Pamukçu, Hatice Eroğlu, Kübra Kalkan Erol, Aysegul Ilhan, Orhan Kocaman

**Affiliations:** ^1^Division of Gastroenterology, Internal Medicine Department, Bezmialem Vakif University, Turkey; ^2^Internal Medicine Department, Şişli Etfal Training and Research Hospital, Turkey; ^3^Sisli Etfal Hamidiye Education and Research Hospital, Clinics of Internal Medicine, Turkey

## Abstract

*Helicobacter pylori* infection and diabetes mellitus are two independent common diseases. It is showed that the worsening glycemic and metabolic control increases the rates of* Helicobacter pylori* infections and* Helicobacter pylori* is shown as one of the common problems in diabetic patients with complaints of gastrointestinal diseases. In this study, we aimed to investigate the prevalence and eradication rates of* Helicobacter pylori* in diabetic patients and the relationship of* Helicobacter pylori* with the risk factors and diabetic complications. In our study, in which we have included 133 patients, we have shown a significant relationship between* Helicobacter pylori* infections and metabolic syndrome, insulin resistance, inflammations, and diabetic complications.

## 1. Introduction

*Helicobacter pylori* (HP) infections are very common worldwide, affecting approximately 50% of the world's population, and are more common especially in developing countries [1]. Although the findings of various studies are inconsistent, the presence of* H. pylori* is found to be higher in diabetic patients compared to nondiabetic patients [2–4].* H. pylori* colonizes in the gastric antrum in all patients, particularly in diabetic patients with impaired metabolic control [2–5]. Beside the well-defined gastric effects of* H. pylori* infection, some studies are also published pointing the extragastric effects of* H. pylori* infection which plays an additional role for the vascular damage in course of atherosclerosis [5, 6].

The presence of* H. pylori* and diabetes mellitus (DM) is one of the main causes of gastrointestinal diseases [2, 7]. Additionally, the presence of* H. pylori* in DM cases plays an important role in the development of gastrointestinal diseases [7]. In particular, the worsening of glycemic and metabolic control increases the incidence of* H. pylori* infections and complaints of dyspepsia [2, 7]. A significant relationship between dyslipidemia and* H. pylori* has been reported. In prospective studies, it has been shown that* H. pylori* eradication improves dyslipidemia and insulin resistance and decreases inflammation [6]. Although the relationship between* H. pylori* infection and DM and the complications secondary to diabetes is not clear, it is known that neuropathy and hyperglycemia play an important role in* H. pylori* colonization in the gastric epithelium [8]. Although the findings of various studies are inconsistent, it has been shown that there is a significant relationship between microvascular complications (nephropathy, neuropathy, and retinopathy) and* H. pylori* [6, 9, 10].

Although there is no clear-cut consensus in the literature, it has been reported that HPeradication is noticeably lower in diabetic patients. There are a limited number of studies on the factors that play a role in HPeradication. We investigated the HP prevalence, HP eradication rates, and confounding factors affecting HP eradication in diabetic patients.

## 2. Materials and Methods

133 dyspeptic patients aged 18 to 65 and recruited from the internal medicine outpatient clinic were included into the study. All patients provided written informed consent to participate. 62 out of 133 patients had Type 2 DM. Exclusion criteria were (1) patients over 65 years; (2) patients having diabetes for more than 5 years; (3) patients with apparent proteinuria, creatinine values of >1.2 mg/dL, triglyceride levels of >400 mg/dL, and HbA1C of >8% with positive urine culture; (4) patients who received ulcer treatment within the last three months; (5) patients currently using proton pump inhibitor or H2 receptor blockers; (6) patients with a history of* H. pylori* eradication treatment; and (7) patients with vascular and inflammatory diseases.

Demographics (age, gender, and duration of disease) of the patients were documented. Height, weight, and waist circumference were measured and BMI was calculated as weight/height^2^ (kg/m^2^). Systolic and diastolic blood pressure measurements using standard sphygmomanometry were performed. Biochemical investigation including hemoglobin A1C (HbA1C), cholesterol, triglyceride, HDL, LDL, fasting blood glucose, leukocyte count, thrombocyte count, erythrocyte sedimentation rate, C reactive protein, and fibrinogen levels was performed in all patients. Approval of the local ethics committee was obtained for the study.

Peripheral vascular disease was assessed based on the presence of intermittent claudication clinically and the absence of pulse in physical examinations. Retinopathy was determined with standard fundus examination. The presence of neuropathy was determined as per abnormal sensorimotor findings in examinations. Patients with a protein amount of 30 to 300 mg in 24-hour urine were considered cases with microalbuminuria, and those with ≥300 mg were considered cases with apparent proteinuria. In order to determine HP infection, the HPantigen stool test was used as it is a useful method for detection of active and repetitive HP infections [11]. The success rate of eradication was checked four weeks after the end of all treatments.

In all patients with dyspeptic complaints, the presence of* H. pylori* was investigated by the* H. pylori* antigen stool test, and the patients were compared by dividing them into two groups (Type 2 DM patients and nondiabetic patients). The relationship between* H. pylori* positivity and demographic clinical anthropometric and inflammatory parameters was investigated in all patients. The relationship between* H. pylori* positivity and retinopathy, nephropathy, neuropathy, and HbA1C was investigated in diabetic patients.

Eradication treatment (clarithromycin, amoxicillin, and omeprazole) was given to* H. pylori* positive patients for two weeks. Additional omeprazole treatment without antibiotics was continued for 4 weeks. Two weeks after cessation of all treatments, eradication rates were determined by stool antigen test and a comparison was made.

### 2.1. Statistical Analysis

SPSS 17.0 package program was used for statistical analysis of data. The data were summarized in percentage, mean ± SD, and median values. The presence of* H. pylori* and eradication of* H. pylori*, as well as the relationship with demographic clinical anthropometric and inflammatory parameters, were analyzed using chi-square test, Fischer's exact test, and independent *T*-sample test. The relationship between the presence of* H. pylori* and eradication of* H. pylori* in diabetic patients was analyzed using chi-square test and Fischer's exact test. The results were assessed with hazard ratio and in a 95% confidence interval. A *P* < 0.05 value was considered statistically meaningful in the analyses.

## 3. Results

*H. pylori* positivity was detected in 64.5% of the diabetic patients and in 43.6% of the control group.* H. pylori* positivity was detected in 53.4% of all patients. The relationship between* H. pylori* positivity and inflammatory, demographic, clinical, and anthropometric parameters was shown in Tables 1 and 2.

When the relationship between the complications in diabetic patients and* H. pylori* positivity was evaluated,* H. pylori* positivity was significantly associated with the presence of nephropathy and neuropathy. Although retinopathy was more common in patients with* H. pylori* positivity, no statistical significance was found between groups. Additionally, a significant relationship between glycemic control and the presence of* H. pylori* was detected. While 48.5% of the patients with HbA1C ≤7 were* H. pylori* positive, 82.8% of the patients with HbA1C >7 were found to be* H. pylori* positive (Table 3).

The rate of* H. pylori* eradication in diabetic and control groups was 62.5% and 93.5%, respectively. The factors affecting the rate of* H. pylori* eradication were shown in Table 4.

When the relationship between the complications in diabetic patients and* H. pylori* eradication was evaluated, a significant relationship between nephropathy and neuropathy with successful eradication of* H. pylori* was detected. Although retinopathy is more common in patients without* H. pylori* eradication, no statistical significance was detected. Additionally, a significant relationship between glycemic control and the presence of* H. pylori* eradication was detected. While* H. pylori* were successfully eradicated in 81.2% of the patients with HbA1C ≤7,* H. pylori* could be eradicated in 50% of the patients with HbA1C (Table 5).

## 4. Discussion

DM patients are usually prone to chronic infections. While the findings of studies on the prevalence of* H. pylori* in DM patients are contradictory, in our study,* H. pylori* positivity was detected in 64.5% of the DM patients and in 43.6% of the control group (*P* < 0.05). While Gentile et al. found that the prevalence of* H. pylori* infection was 74.4% in DM patients and 50% in the control group (*P* < 0.01), Gentile et al. found that the prevalence of* H. pylori* was significantly higher in the DM group compared to the control group [12, 13]. However, some studies did not find any significant difference in the DM group and the control group with regard to* H. pylori* infections [9, 14]. It is well known that diabetic patients are prone to chronic infections because of cellular and humoral immune deficiency. As a result of delayed gastric emptying due to gastroparesis diabeticorum, bacterial overgrowth occurs and this poses a risk for* H. pylori* infections. Achlorhydria and reduced acid secretion are a negative factor for* H. pylori* infections. Additionally, leukocyte dysfunction and hyperglycemia are a predisposing factor for infections and facilitate secondary* H. pylori* colonization to antibiotics taken. Based on all these factors, it can be said that the prevalence of* H. pylori* is increased in diabetics [9].

Studies report that HP infections cause microvascular damage and trigger premature development of atherosclerosis in patients [5, 6]. Although the underlying mechanism is not fully known, there are many findings which support this [5]. First of all, it has been shown that HP plays a role in the thickening of the intima media, atherosclerotic plaque destabilization, and atherosclerotic plaque development after vessel wall invasion [5, 15]. Due to vessel wall invasion of the bacteria, an increase in maturation and activation of monocytes and an increase in the proliferation of smooth muscle or endothelial cells occur, and, as a result, thrombosis and ischemia develop. It is thought that the endotoxins produced by the bacteria play a role in the maturation of monocytes. Activation of monocytes leads to the production of inflammatory cytokines, after which it triggers platelet aggregation and procoagulant activity [16]. Secondly, atherosclerotic plaques due to HP trigger high amounts of IL-6 and TNF-*α*. An increase in these cytokines causes endothelial dysfunction and insulin resistance. While IL-6 increases the production of hepatic gluconeogenesis and triglycerides, TNF-*α* modifies the lipid levels by inhibiting the lipoprotein lipase activity and activating hepatic lipogenesis [17]. Thirdly, the inflammation secondary to the plaque which forms due to HP triggers the peroxidation of membrane lipids, oxidation of LDL cholesterol, antioxidant loss, an increase in production of various superoxidases, and activation of macrophages, T-lymphocytes, and lipoprotein-a [18]. Additionally, chronic HP infection causes atrophic gastritis, and, as a result of this, it reduces absorption of folate and B12. B12 and folate are required for conversion of homocysteine to methionine. However, the homocysteine level increases due to the deficiency of B12 and folate in HP infections. Homocysteine plays a role in vascular endothelial damage and increases atherogenesis and thrombogenesis. It also increases platelet functions, coagulation, and LDL oxidation [19]. Similar to our study, many other studies have shown that, in the presence of HP infection, inflammatory indicators increase significantly [5, 20]. It has been reported that, as a result of an increase in inflammation due to HP infection and the triggering of arthrosclerosis, insulin resistance and metabolic syndrome increase, and the complications also increase in diabetic patients. A study on the Japanese population has shown that metabolic syndrome increases in HP infection [21]. Gunji et al. have shown in their study that HP infection significantly increases insulin resistance in asymptomatic patients [22]. Similar to the said studies, our study has also shown that metabolic syndrome significantly increases in patients with HP infection. Hamed et al. have shown in their study that, in HP-positive diabetic patients, the prevalence of macrovascular (cardiovascular and cerebrovascular diseases) and microvascular (nephropathy, neuropathy, and retinopathy) complications is significantly higher [5]. Furthermore, there are studies which report that microalbuminuria is significantly higher in HP-positive patients, regardless of the development of diabetes [6]. Demir et al. have reported that there is a significant relationship between HP-positivity and neuropathy, but there is no significant relationship between HP-positivity and retinopathy and neuropathy [9]. Similar to the said studies, our study has found a significant relationship between HP-positivity and neuropathy but has not found a significant relationship between HP-positivity and retinopathy.

HP eradication is more difficult in diabetic patients. Sargýn et al. attained 50% HP eradication in DM patients and 85% HP eradication in non-DM patients [7]. Zojaji et al. attained successful eradication in 62% of DM patients, and a significant relationship was found between eradication and HbA1C [23]. In a study by Tseng, it was reported that comorbid conditions such as obesity, dyslipidemia, retinopathy, and neuropathy were significantly associated with HP eradication [10]. Our study has also shown that HP eradication is significantly lower in diabetic patients with retinopathy and neuropathy and with impaired glycemic and metabolic control. The fact that the HP eradication percentage is low in diabetic patients can be explained by the immunosuppressive condition in DM patients. Additionally, frequent bacterial and mycotic infections and the development of resistance due to drug use also play an important role [24].

As a result, we have shown in our study that there is a significant relationship between HP infections and metabolic syndrome, insulin resistance, inflammations, and diabetic complications. We have also shown that similar parameters are effective in HP eradication. In the light of the studies, we think that patients with impaired metabolic and glycemic control should be treated in case of HP infection.

## Figures and Tables

**Table 1:** The relationship between *H*. *pylori* positivity and inflammatory parameters.

Variables	Total	*H*. *pylori* (+) subjects	*H*. *pylori* (−) subjects	*P* value
CRP^*^	4.8 ± 3.4	5.5 ± 3.9	3.9 ± 2.3	<0.05
Leukocytes	7.0 ± 1.8	7.1 ± 1.6	6.9 ± .9	NS
Thrombocytes	250 ± 65	252 ± 69	248 ± 61	NS
Sedimentation^*^	9.4 ± 8.2	11.3 ± 8.9	7.3 ± 6.8	<0.05
Ferritin^*^	86.5 ± 91.7	122 ± 105	45.6 ± 47	<0.05
Fibrinogen^*^	321 ± 77	336 ± 82	303 ± 67	<0.05

^*^Significant variables.

**Table 2:** The relationship between *H*. *pylori* positivity and demographic, clinical, and anthropometric parameters.

Variables	Total	*H*. *pylori* (+) subjects	*H*. *pylori* (−) subjects	*P* value
Gender				
Female	70	34 (48.5%)	36 (51.5%)	NS
Male	63	37 (58.7%)	26 (41.3%)	NS
Age	47 ± 12	48.7 ± 12.1	46.9 ± 13.1	NS
Body mass index				NS
Normal	30	12 (40%)	18 (60%)	NS
Overweight	53	31 (58.4%)	22 (41.6%)	NS
Obese	50	28 (56%)	22 (44%)	NS
Waistline^*^	94 ± 11	95.7 ± 10	91.9 ± 11.8	<0.05
Hypertension				
Yes^*^	49	34 (69.3%)	15 (30.7%)	<0.05
No	84	37 (44%)	47 (56%)	NS
Cholesterol				NS
≥200	67	38 (56.7%)	29 (43.3%)	NS
<200	66	33 (50%)	33 (50%)	NS
Triglyceride				
≥150^*^	42	30 (71.4%)	12 (28.6%)	<0.05
<150	91	41 (45%)	50 (55%)	NS
Low density lipoprotein				
≥100	87	51 (58.6%)	36 (41.4%)	NS
<100	46	20 (43.4%)	26 (56.6%)	NS
High density lipoprotein				
Male ≥40, female ≥50	98	47 (%47.9)	51 (52.1%)	NS
Male <40, female <50^*^	35	24 (68.5%)	11 (31.5%)	<0.05
Glucose^*^	106 ± 35	117 ± 41	93 ± 21	<0.05
Creatinine	0.8 ± 0.2	0.84 ± 0.23	0.78 ± 0.19	NS
Patient groups				
DM^*^	62	40 (64.5%)	20 (35.5%)	<0.05
Non-DM	71	31 (43.6%)	40 (56.7%)	NS

^*^Significant variables.

**Table 3:** The relationship between the complications in diabetic patients and *H*. *pylori* positivity.

Variables	Total DM	*H*. *pylori* (+) subjects	*H*. *pylori* (−) subjects	*P* value
Nephropathy				
Yes^*^	23	21 (91.3%)	2 (8.7%)	<0.05
No	39	19 (48.7%)	20 (51.3%)	
Neuropathy				
Yes^*^	29	24 (82.8%)	5 (18.2%)	<0.05
No	33	16 (48.4%)	17 (51.6%)	
Retinopathy				
Yes	20	14 (70%)	6 (30%)	NS
No	42	24 (57.1%)	18 (42.9%)	
HbA1C				
6-7^*^	33	16 (48.5%)	17 (51.5%)	<0.05
7-8	29	24 (82.8%)	5 (17.2%)	

^*^Significant variables.

**Table 4:** The factors affecting the rate of *H*. *pylori* eradication.

Variables	Total	Those eradicated	Those not eradicated	*P* value
Gender				
Female	34	25 (73.5%)	9 (26.5%)	NS
Male	37	29 (78.3%)	8 (21.7%)	
Age	48.7 ± 12.1	48.1 ± 13.3	50.8 ± 7.2	NS
Body mass index				
Normal	12	12 (100%)	0 (0%)	
Overweight	31	28 (90.3%)	3 (9.7%)	
Obese^*^	28	14 (50%)	14 (50%)	<0.05
Waistline^*^	95.7 ± 10	94.3 ± 9.8	100 ± 9.4	<0.05
Hypertension				
Yes^*^	34	22 (64.7%)	12 (35.3%)	<0.05
No	37	32 (86.4%)	5 (13.6%)	
Cholesterol				
≥200^*^	38	24 (63.1%)	14 (36.9%)	<0.05
<200	33	30 (90.9%)	3 (9.1%)	
Triglyceride				
≥150^*^	30	19 (63.3%)	11 (36.7%)	<0.05
<150	41	35 (85.3%)	6 (14.7%)	
Low density lipoprotein				NS
≥100	51	37 (72.5%)	14 (27.5%)	
<100	20	17 (85%)	3 (15%)	
High density lipoprotein				NS
Male ≥40, female ≥50	47	38 (80.8%)	9 (19.2%)	
Male <40, female <50	24	16 (66.6%)	8 (33.4%)	
Glucose^*^	117 ± 42	110 ± 39	140 ± 41	<0.05
Patient groups				
DM^*^	40	25 (62.5%)	15 (37.5%)	<0.05
Non-DM	31	29 (93.5%)	2 (6.5%)	

^*^Significant variables.

**Table 5:** The relationship between the complications in diabetic patients and *H*. *pylori* eradication.

Variables	Total DM	Those eradicated	Those not eradicated	*P* value
Nephropathy				
Yes^*^	22	10 (45.4%)	12 (54.6%)	<0.05
No	18	15 (83.8%)	3 (16.7%)	
Neuropathy				
Yes^*^	24	11 (45.8%)	13 (54.2%)	<0.05
No	16	14 (87.5%)	2 (22.5%)	
Retinopathy				
Yes	16	9 (56%)	7 (44%)	NS
No	24	16 (66.6%)	8 (33.4%)	
HbA1C				
6-7^*^	16	13 (81.2%)	3 (18.8%)	<0.05
7-8	24	12 (50%)	12 (50%)	

^*^Significant variables.

## References

[1] Pounder R. E., Ng D. (1995). The prevalence of *Helicobacter pylori* infection in different countries. *Alimentary Pharmacology & Therapeutics*.

[2] Devrajani B. R., Shah S. Z. A., Soomro A. A., Devrajani T. (2010). Type 2 diabetes mellitus: a risk factor for *Helicobacter pylori* infection: a hospital based case-control study. *International Journal of Diabetes in Developing Countries*.

[3] Vafaeimanesh J., Parham M., Seyyedmajidi M., Bagherzadeh M. (2014). *Helicobacter pylori* infection and insulin resistance in diabetic and nondiabetic population. *Scientific World Journal*.

[4] Talebi-Taher M., Mashayekhi M., Hashemi M. H., Bahrani V. (2012). *Helicobacter pylori* in diabetic and non-diabetic patients with dyspepsia. *Acta Medica Iranica*.

[5] Hamed S. A., Amine N. F., Galal G. M. (2008). Vascular risks and complications in diabetes mellitus: the role of *Helicobacter pylori* infection. *Journal of Stroke and Cerebrovascular Diseases*.

[6] Chung G. E., Heo N. J., Park M. J., Chung S. J., Kang H. Y., Kang S. J. (2013). *Helicobacter pylori* seropositivity in diabetic patients is associated with microalbuminuria. *World Journal of Gastroenterology*.

[7]  Sargin M., Uygur-Bayramiçli O., Sargýn H., Orbay E., Yavuzer D., Yayla A. (2003). Type 2 diabetes mellitus affects eradication rate of *Helicobacter pylori*. *World Journal of Gastroenterology*.

[8] Talley N. J., Young L., Bytzer P. (2001). Impact of chronic gastrointestinal symptoms in diabetes mellitus on health-related quality of life. *The American Journal of Gastroenterology*.

[9] Demir M., Gokturk H. S., Ozturk N. A., Kulaksizoglu M., Serin E., Yilmaz U. (2008). *Helicobacter pylori* prevalence in diabetes mellitus patients with dyspeptic symptoms and its relationship to glycemic control and late complications. *Digestive Diseases and Sciences*.

[10] Tseng C.-H. (2012). Diabetes, insulin use and *Helicobacter pylori* eradication: a retrospective cohort study. *BMC Gastroenterology*.

[11] Ni Y.-H., Lin J.-T., Huang S.-F., Yang J.-C., Chang M.-H. (2000). Accurate diagnosis of *Helicobacter pylori* infection by stool antigen test and 6 other currently available tests in children. *Journal of Pediatrics*.

[12] Gentile S., Turco S., Oliviero B., Torella R. (1998). The role of autonomic neuropathy as a risk factor of *Helicobacter pylori* infection in dyspeptic patients with type 2 diabetes mellitus. *Diabetes Research and Clinical Practice*.

[13] Marrollo M., Latella G., Melideo D. (2001). Increased prevalence of *Helicobacter pylori* in patients with diabetes mellitus. *Digestive and Liver Disease*.

[14] Anastasios R., Goritsas C., Papamihail C., Trigidou R., Garzonis P., Ferti A. (2002). *Helicobacter pylori* infection in diabetic patients: prevalence and endoscopic findings. *European Journal of Internal Medicine*.

[15] Franceschi F., Sepulveda A. R., Gasbarrini A. (2002). Cross-reactivity of anti-CagA antibodies with vascular wall antigens: possible pathogenic link between *Helicobacter pylori* infection and atherosclerosis. *Circulation*.

[16] Bliss C. M., Golenbock D. T., Keates S., Linevsky J. K., Kelly C. P. (1998). *Helicobacter pylori* lipopolysaccharide binds to CD14 and stimulates release of interleukin-8, epithelial neutrophil-activating peptide 78, and monocyte chemotactic protein 1 by human monocytes. *Infection and Immunity*.

[17] Crabtree J. E., Shallcross T. M., Heatley R. V., Wyatt J. I. (1991). Mucosal tumour necrosis factor *α* and interleukin-6 in patients with *Helicobacter pyloris* associated gastritis. *Gut*.

[18] Kratnov A. E., Pavlov O. N. (2004). *Helicobacter pylori* infection and state of antioxidant protection in patients with the unstable course of ischemic disease. *Eksperimental'naia i Klinicheskaia Gastroenterologiia*.

[19] Harker L. A., Ross R., Slichter S. J. (1976). Homocystine induced arteriosclerosis. The role of endothelial cell injury and platelet response in its genesis. *The Journal of Clinical Investigation*.

[20] Majka J., Róg T., Konturek P. C. (2002). Influence of chronic *Helicobacter pylori* infection on ischemic cerebral stroke risk factors. *Medical Science Monitor*.

[21] Gunji T., Matsuhashi N., Sato H. (2008). *Helicobacter pylori* infection is significantly associated with metabolic syndrome in the Japanese population. *American Journal of Gastroenterology*.

[22] Gunji T., Matsuhashi N., Sato H. (2009). *Helicobacter pylori* infection significantly increases insulin resistance in the asymptomatic Japanese population. *Helicobacter*.

[23] Zojaji H., Ataei E., Sherafat S. J., Ghobakhlou M., Fatemi S. R. (2013). The effect of the treatment of *Helicobacter pylori* infection on the glycemic control in type 2 diabetes mellitus. *Gastroenterology and Hepatology from Bed to Bench*.

[24] Zweet V., Megraud F. (1998). Diagnosis. *Current Opinion in Gastroenterology*.

